# Supplementary material for: Consciousness-specific dynamic interactions of brain integration and functional diversity
Source: Nat Commun. 2019 Oct 10;10:4616. doi: 10.1038/s41467-019-12658-9 (PMC6787094; doi:10.1038/s41467-019-12658-9)
Supplement: Supplementary file 1 — Supplementary Information [file 41467_2019_12658_MOESM1_ESM.pdf]

Supplementary Information for Consciousness-specific dynamic interactions of brain integration and functional diversity

Luppi et al.

September 7, 2019

Supplementary Figures

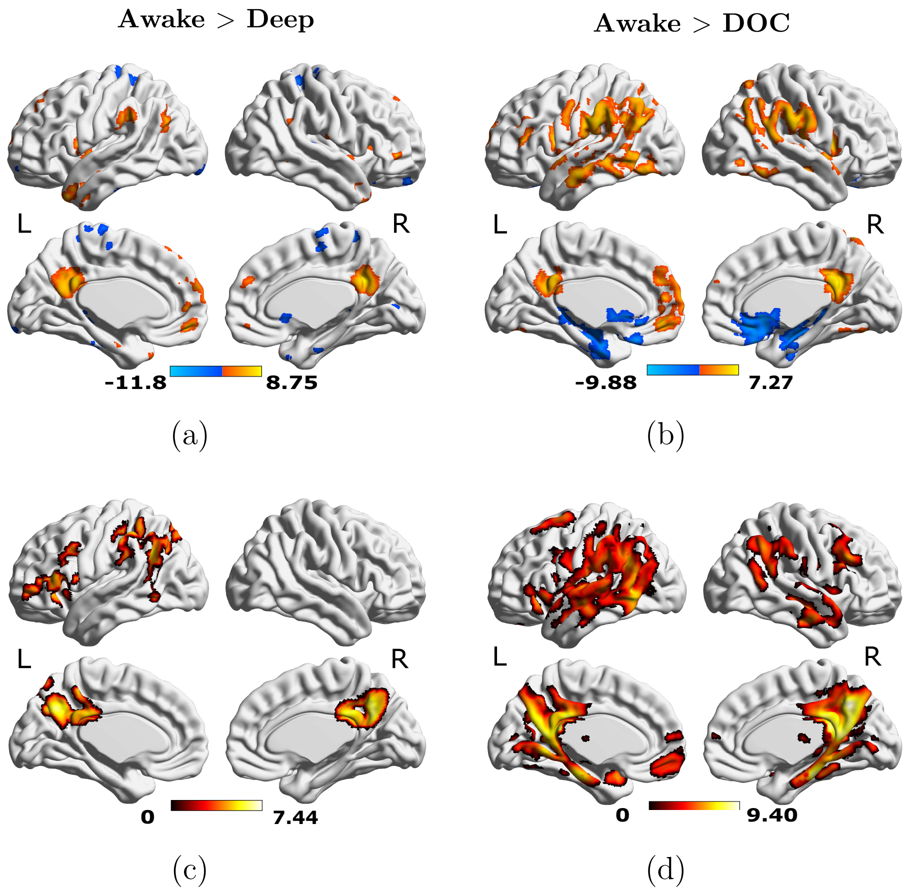

Figure 1: Brain maps of consciousness-related differences in sample entropy and Intrinsic Connectivity Contrast. (a and b) Significant differences (t-value) in Intrinsic Connectivity as a result of propofol anaestesia (Awake - Deep, left) and brain injury (Awake - DOC, right). (c and d) Significant differences (t-value) in the sample entropy of BOLD timeseries as a result of propofol anaestesia (Awake - Deep, left) and brain injury (Awake - DOC, right). Images are displayed on a standard Montreal Neurological Institute (MNI-152) structural T1 scan, in neurological convention (L is L). Maps of t-values are thresholded at uncorrected $p < 0.001$ at the voxel level, with further FWE cluster correction to achieve $p < 0.05$ at the cluster level (repeated-measures t-test for Awake > Deep, and two-samples t-test for Awake > DOC).

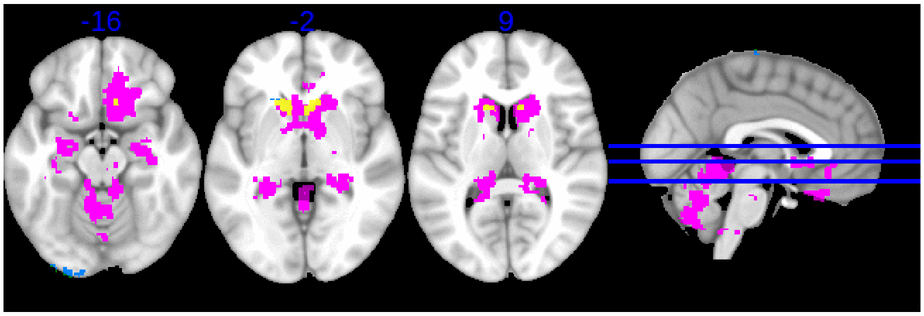

Figure 2: Overlaps (yellow) of significant increases of Intrinsic Connectivity Contrast in anaesthesia (turquoise) and DOC (magenta), shown on multiple axial slices of a standard Montreal Neurological Institute (MNI-152) structural T1 scan, in neurological convention, visualised using MRIcron software (https://www.nitrc.org/projects/mricron). Location of each axial slice is identified on the midline sagittal section displayed on the right, with the corresponding coordinate in the Z plane shown above each axial slice.

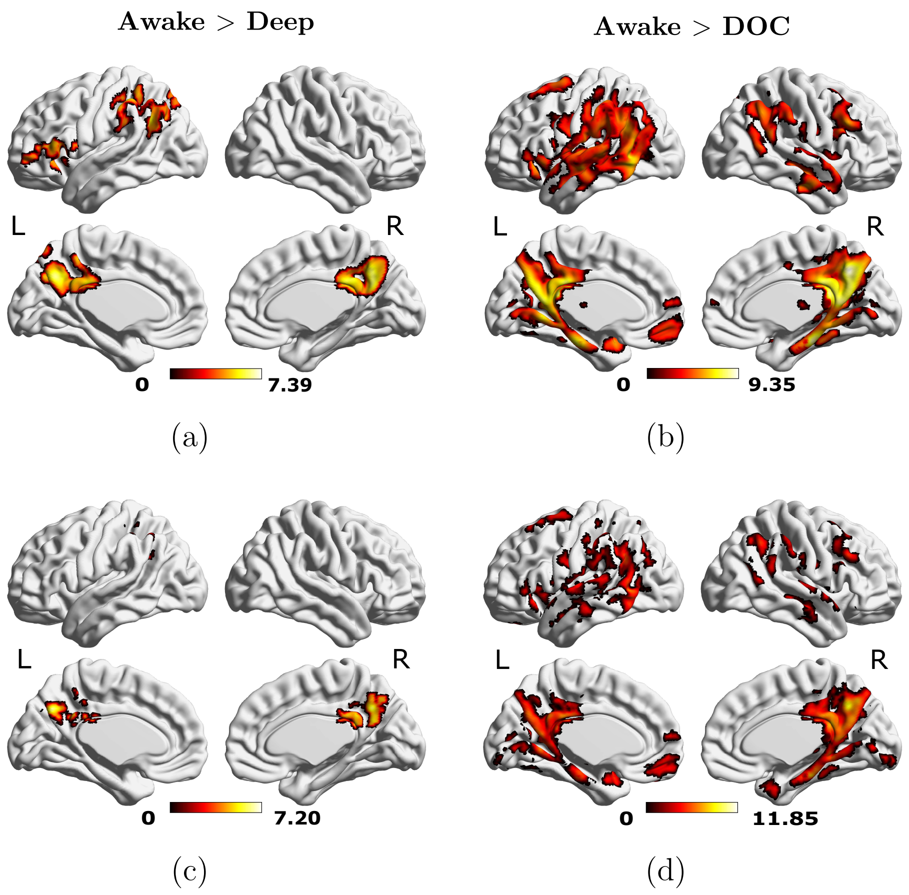

Figure 3: Brain maps of consciousness-related differences in Sample Entropy demonstrating robustness to use of alternative parameters. (a and b) Significant differences in sample entropy as a result of propofol anaestesia (Awake - Deep) calculated with $m = 2$ and $r = 0.3$ times the standard deviation of the data, as in [1], and subsequent 10mm FWHM smoothing. (c and d) Significant differences in sample entropy (with $m = 3$ and $r = 0.6$ standard deviations) as a result of propofol anaestesia (Awake - Deep) and brain injury (Awake - DOC, right), applying 6mm FWHM smoothing. Maps of t-values (thresholded at uncorrected $p < 0.001$ at the voxel level, with further FWE cluster correction to achieve $p < 0.05$ at the cluster level; repeated-measures t-test for Awake > Deep, and two-samples t-test for Awake > DOC) are displayed on medial and lateral surfaces of a standard Montreal Neurological Institute (MNI-152) structural T1 scan, in neurological convention (L is L).

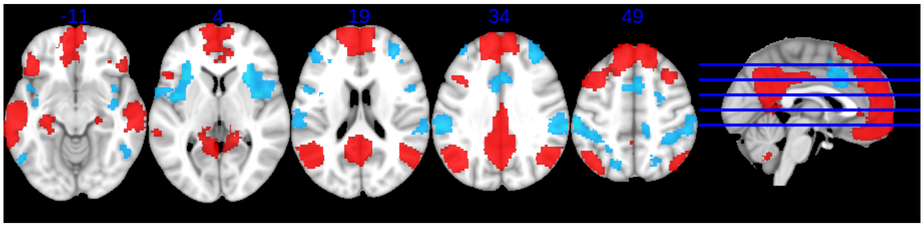

(a)

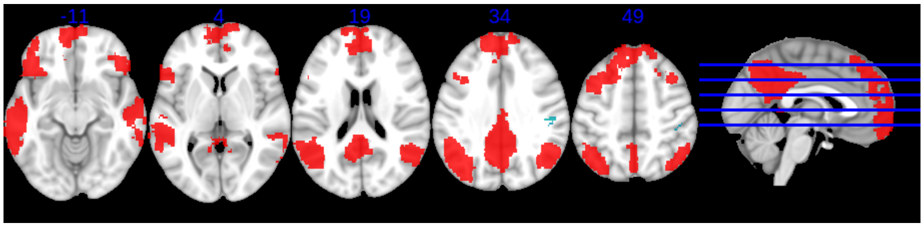

(b)

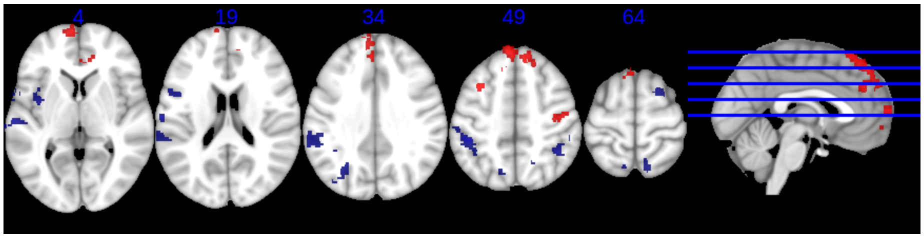

(c)

Figure 4: Results of the follow-up analysis using the left angular gyrus cluster as seed region to investigate the nature of intrinsic connectivity alterations, for the propofol dataset. (a) awake healthy volunteers; (b) the same individuals under deep propofol anaesthesia. Positive correlations shown in red, negative correlations in blue. (c) significant differences in connectivity between the two conditions: conscious > unconscious (red) and unconscious > conscious (blue). Results are shown on multiple axial slices of a standard Montreal Neurological Institute (MNI-152) structural T1 scan, in neurological convention, visualised using MRIcron software (https://www.nitrc.org/projects/mricron). Location of each axial slice is identified on the midline sagittal section displayed on the right, with the corresponding coordinate in the Z plane shown above each axial slice. Maps are thresholded at uncorrected $p < 0.001$ at the voxel level, with further FWE cluster correction to achieve $p < 0.05$ at the cluster level (repeated-measures t-test).

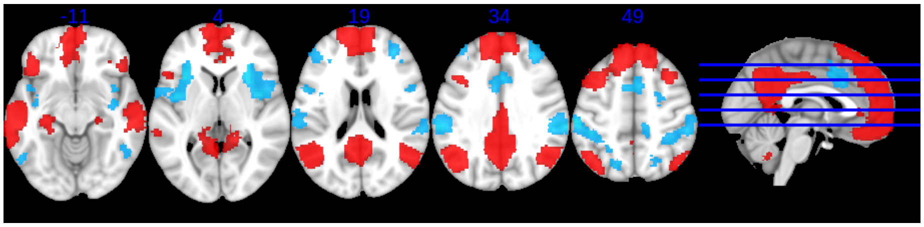

(a)

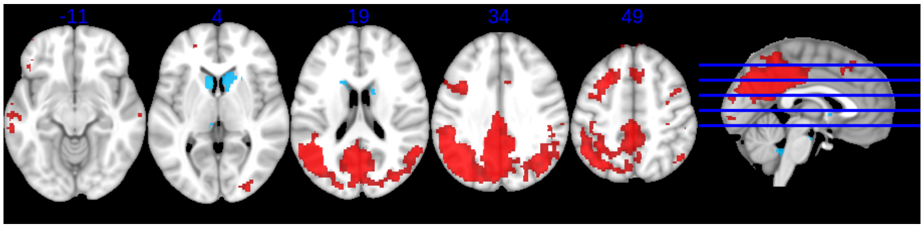

(b)

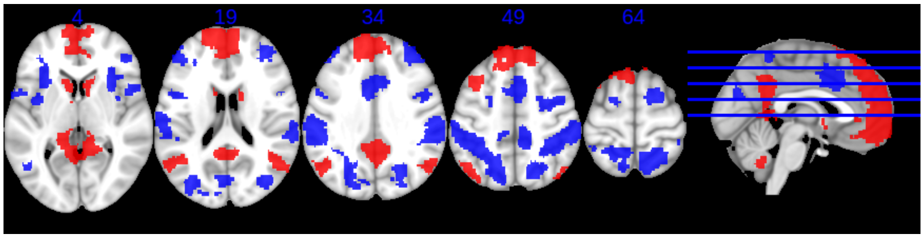

(c)

Figure 5: Results of the follow-up seed-based connectivity analysis using the left angular gyrus cluster as seed region to investigate the nature of intrinsic connectivity alterations, for the DOC dataset. (a) awake healthy volunteers; (b) DOC patients. Positive correlations shown in red, negative correlations in blue. (c) significant differences in connectivity between the two conditions: conscious $>$ unconscious (red) and unconscious $>$ conscious (blue). Results are shown on multiple axial slices of a standard Montreal Neurological Institute (MNI-152) structural T1 scan, in neurological convention, visualised using MRIcron software (https://www.nitrc.org/projects/mricron). Location of each axial slice is identified on the midline sagittal section displayed on the right, with the corresponding coordinate in the Z plane shown above each axial slice. Maps are thresholded at uncorrected $p < 0.001$ at the voxel level, with further FWE cluster correction to achieve $p < 0.05$ at the cluster level (two-samples t-test).

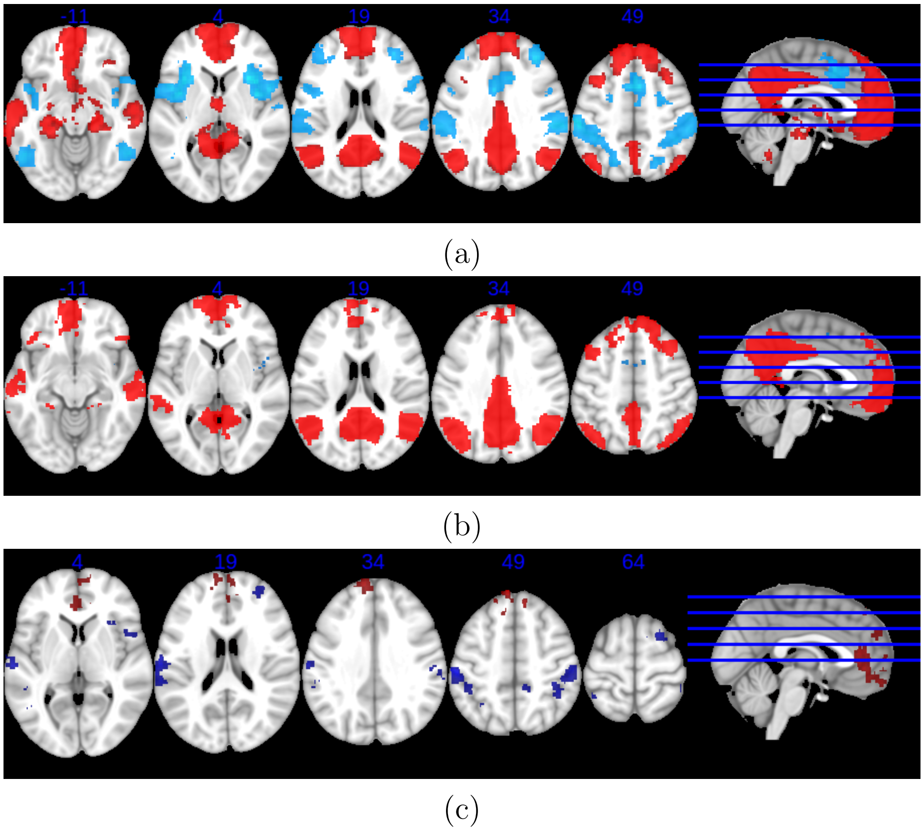

Figure 6: Results of the follow-up seed-based connectivity analysis using the posterior cingulate/precuneus cluster as seed region to investigate the nature of intrinsic connectivity alterations, for the propofol dataset. (a) awake healthy volunteers; (b) the same individuals under deep propofol anaesthesia. Positive correlations shown in red, negative correlations in blue. (c) significant differences in connectivity between the two conditions: conscious > unconscious (red) and unconscious > conscious (blue). Results are shown on multiple axial slices of a standard Montreal Neurological Institute (MNI-152) structural T1 scan, in neurological convention, visualised using MRIcron software (https://www.nitrc.org/projects/mricron). Location of each axial slice is identified on the midline sagittal section displayed on the right, with the corresponding coordinate in the Z plane shown above each axial slice. Maps are thresholded at uncorrected $p < 0.001$ at the voxel level, with further FWE cluster correction to achieve $p < 0.05$ at the cluster level (repeated-measures t-test).

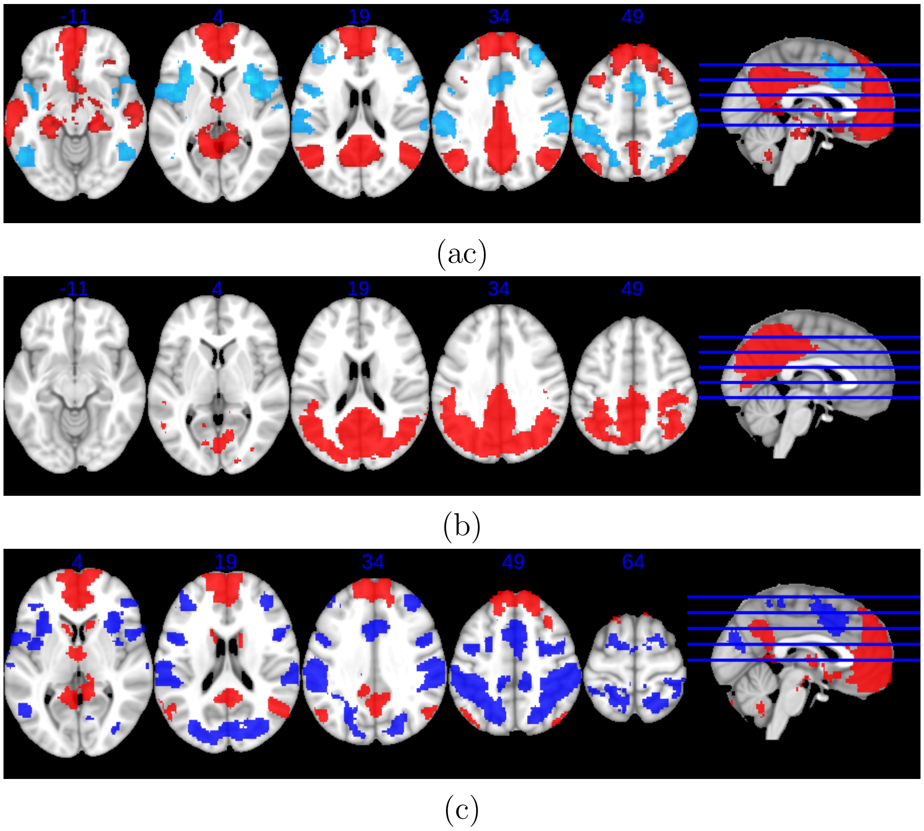

Figure 7: Results of the follow-up seed-based connectivity analysis using the posterior cingulate/precuneus cluster as seed region to investigate the nature of intrinsic connectivity alterations, for the DOC dataset. (a) awake healthy volunteers; (b) DOC patients. Positive correlations shown in red, negative correlations in blue. (c) significant differences in connectivity between the two conditions: conscious > unconscious (red) and unconscious > conscious (blue). Results are shown on multiple axial slices of a standard Montreal Neurological Institute (MNI-152) structural T1 scan, in neurological convention, visualised using MRIcron software (https://www.nitrc.org/projects/mricron). Location of each axial slice is identified on the midline sagittal section displayed on the right, with the corresponding coordinate in the Z plane shown above each axial slice. Maps are thresholded at uncorrected $p < 0.001$ at the voxel level, with further FWE cluster correction to achieve $p < 0.05$ at the cluster level (two-samples t-test).

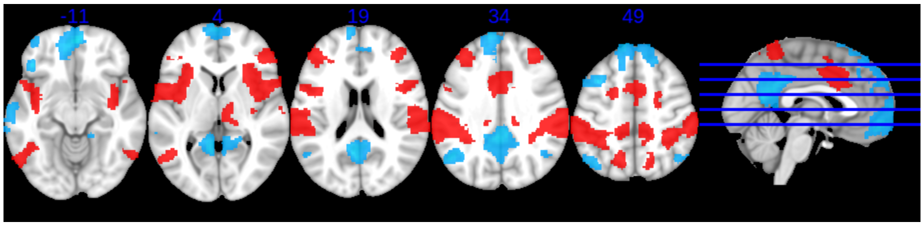

(a)

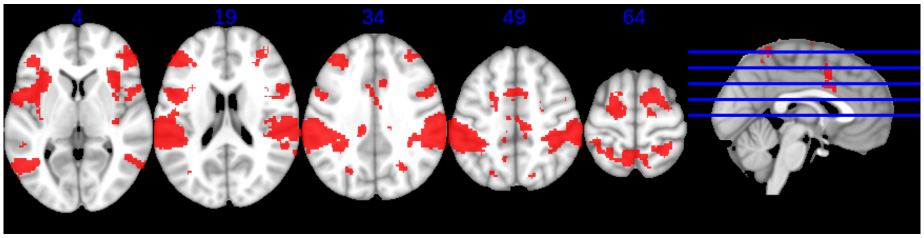

(b)

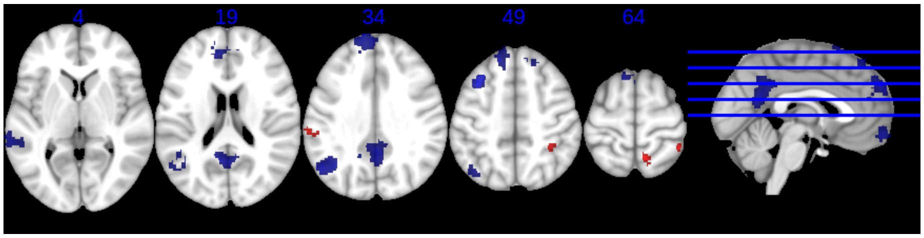

(c)

Figure 8: Results of the follow-up seed-based connectivity analysis using the left supramarginal gyrus cluster as seed region to investigate the nature of intrinsic connectivity alterations, for the propofol dataset. (a) awake healthy volunteers (b) the same individuals under deep propofol anaesthesia. Positive correlations shown in red, negative correlations in blue. (c) significant differences in connectivity between the two conditions: conscious > unconscious (red) and unconscious > conscious (blue). Results are shown on multiple axial slices of a standard Montreal Neurological Institute (MNI-152) structural T1 scan, in neurological convention, visualised using MRIcron software (https://www.nitrc.org/projects/mricron). Location of each axial slice is identified on the midline sagittal section displayed on the right, with the corresponding coordinate in the Z plane shown above each axial slice. Maps are thresholded at uncorrected $p < 0.001$ at the voxel level, with further FWE cluster correction to achieve $p < 0.05$ at the cluster level (repeated-measures t-test).

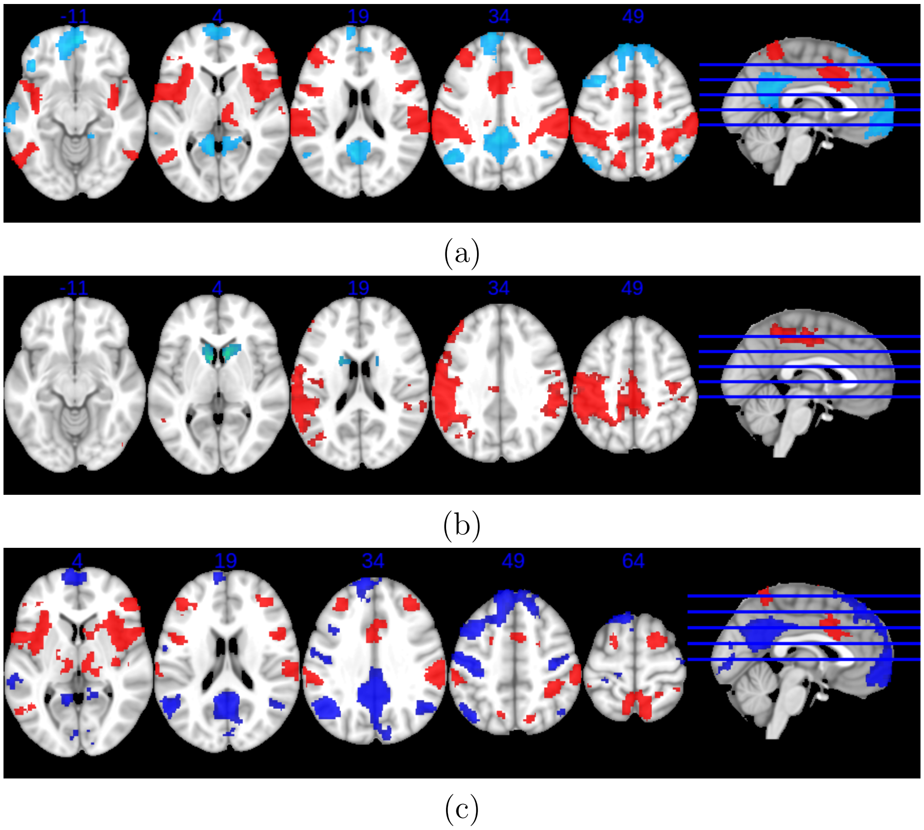

Figure 9: Results of the follow-up seed-based connectivity analysis using the left supramarginal gyrus cluster as seed region to investigate the nature of intrinsic connectivity alterations, for the DOC dataset. (a) awake healthy volunteers; (b) DOC patients. Positive correlations shown in red, negative correlations in blue. (c) significant differences in connectivity between the two conditions: conscious $>$ unconscious (red) and unconscious $>$ conscious (blue). Results are shown on multiple axial slices of a standard Montreal Neurological Institute (MNI-152) structural T1 scan, in neurological convention, visualised using MRIcron software (https://www.nitrc.org/projects/mricron). Location of each axial slice is identified on the midline sagittal section displayed on the right, with the corresponding coordinate in the Z plane shown above each axial slice. Maps are thresholded at uncorrected $p < 0.001$ at the voxel level, with further FWE cluster correction to achieve $p < 0.05$ at the cluster level (two-samples t-test).

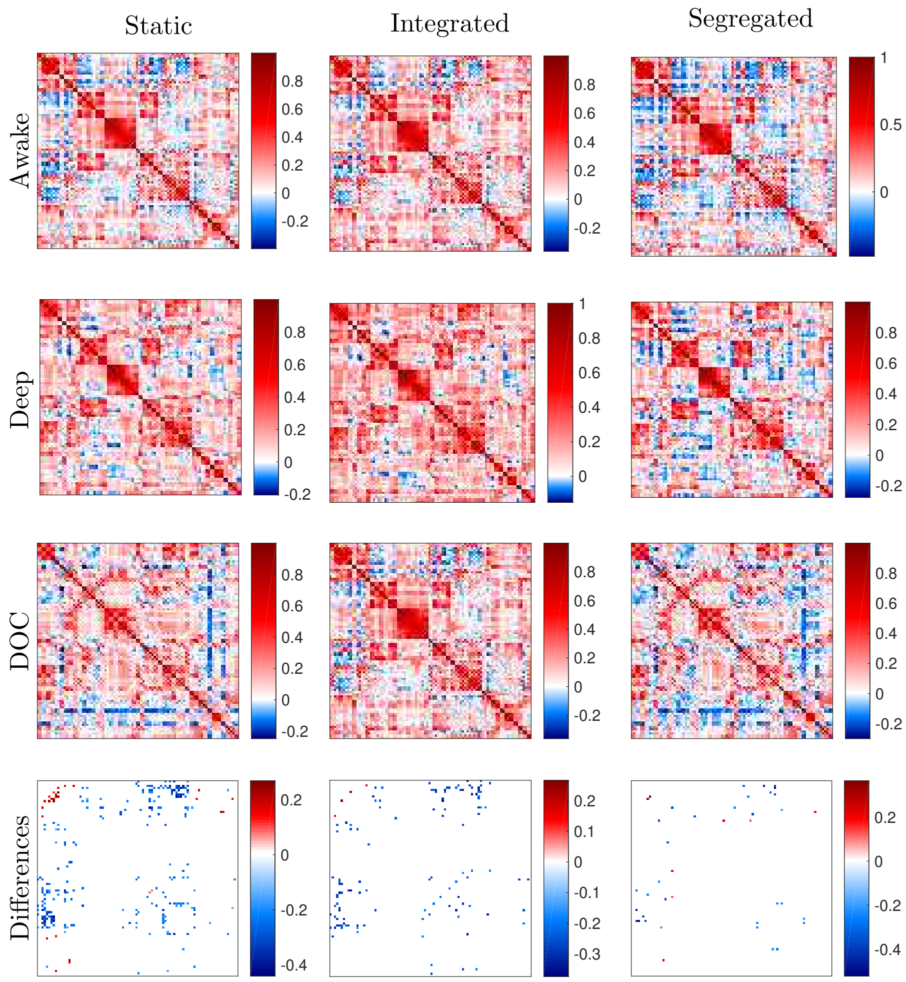

Figure 10: Group-averaged functional connectivity matrices for the static, integrated and segregated states, for awake healthy controls (top), anaesthetised individuals, and DOC patients. Red indicates positive value of Pearson correlation, and blue indicates a negative value (anticorrelation). The bottom row shows differences between conscious and unconscious FC matrices that were present both in anaesthesia (repeated-measures t-test) and DOC (two-samples t-test, FDR-corrected). For integrated and segregated states, differences that were present in both states are not shown, in order to emphasise state-specific ones. Here, red indicates a common positive difference (conscious > unconscious), and blue a common negative difference (unconscious > conscious). Each matrix has been reordered so that ROIs belonging to the same resting-state network [2] are adjacent to each other, in the following order: Default mode, visual, somatomotor, salience, dorsal attention, fronto-parietal and limbic. Source data are provided as a Source Data file.

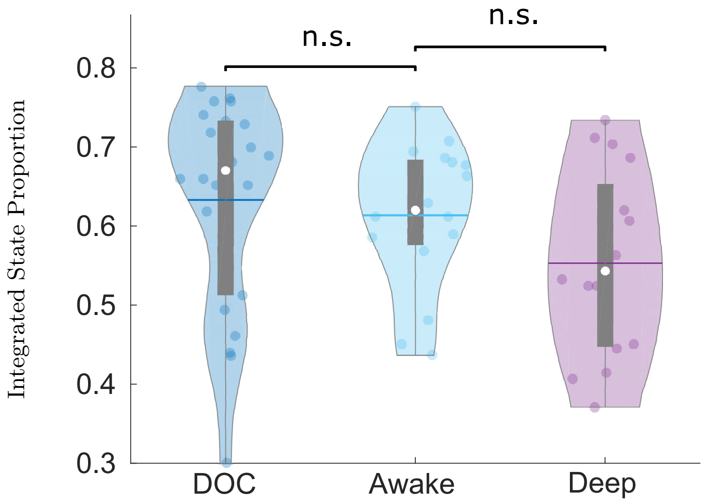

Figure 11: Violin plots of the of the proportion of time spent in the predominantly integrated state (calculated as the number of dynamic functional connectivity matrices of each individual that were assigned to the cluster corresponding to higher integration, over the total number of dynamic matrices), comparing conscious healthy controls and unconscious individuals due to anaesthesia (repeated-measures t-tests) and brain injury (two-samples t-tests). * n.s. not significant; white circle, mean; center line, median; box limits, upper and lower quartiles; whiskers, 1.5x interquartile range. Source data are provided as a Source Data file.

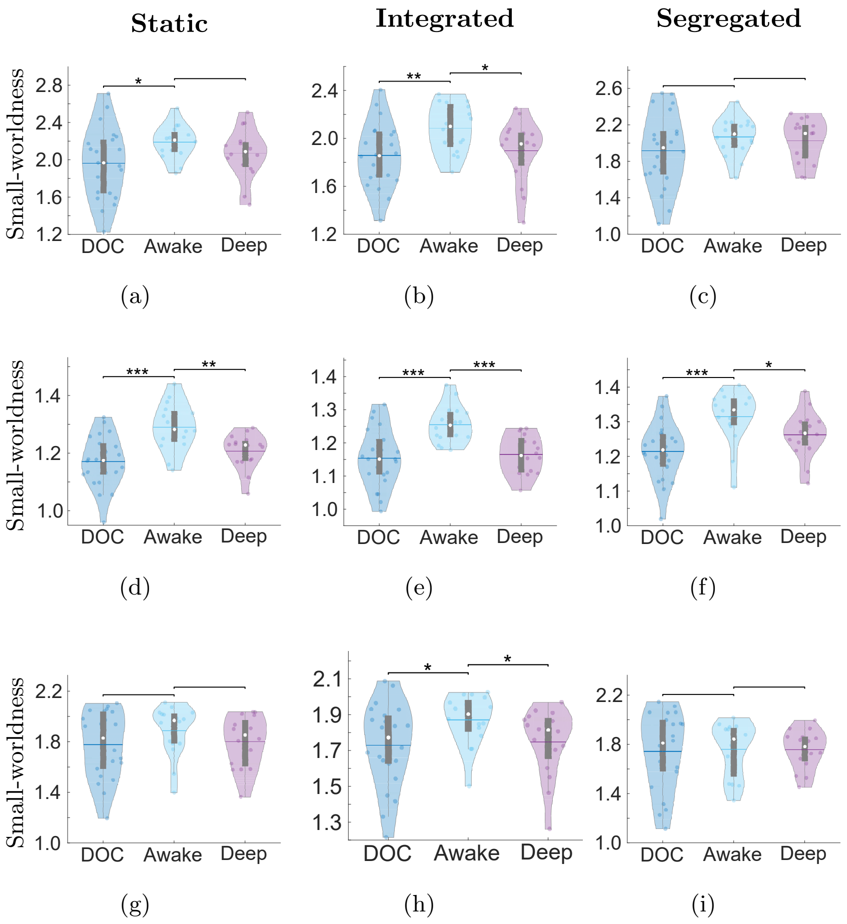

Figure 12: Violin plots of the small-world measure of brain networks based on different network definitions for the static (left), integrated (middle) and segregated (right) states of functional connectivity, comparing conscious healthy controls and unconscious individuals due to anaesthesia (repeated-measures t-tests) and brain injury (two-samples t-tests). Top row: network based on the AAL 90-ROI atlas, binarised and thresholded between 10 and 25%. Middle row: network based on the AAL 90-ROI atlas, weighted and thresholded between 30 and 50%. Bottom row: network based on the Lausanne 234-ROI atlas, weighted and thresholded between 10 and 25%. For each network type, plots show the average over the range of thresholds considered. The small-world index was calculated as the ratio of normalised clustering coefficient to normalised characteristic path length. * $p < 0.05$; ** $p < 0.01$; *** $p < 0.001$; white circle, mean; center line, median; box limits, upper and lower quartiles; whiskers, 1.5x interquartile range. Source data are provided as a Source Data file.

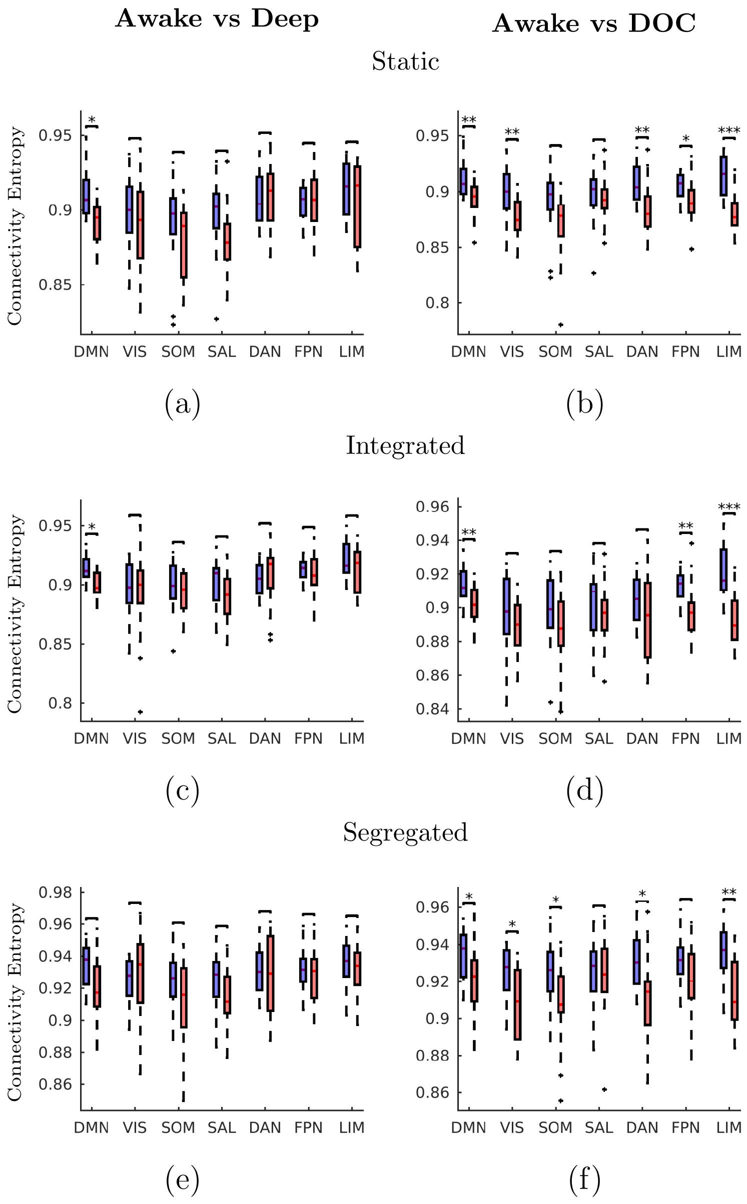

Figure 13: Boxplots of the mean connectivity entropy for the static, integrated and segregated states, comparing conscious healthy controls (blue) and unconscious individuals (red) due to anaesthesia (left panels; repeated-measures t-tests, FDR-corrected) and brain injury (right panels; two-samples t-tests, FDR-corrected) for each of seven well-known resting-state networks [2]. DMN, default mode network; VIS, visual network; SOM, somatomotor network; SAL, salience network; DAN, dorsal attention network; FPN, fronto-parietal network; LIM, limbic network. * $p < 0.05$; ** $p < 0.01$; *** $p < 0.001$; center line, median; box limits, upper and lower quartiles; whiskers, 1.5x interquartile range; plus signs, outliers. Source data are provided as a Source Data file.

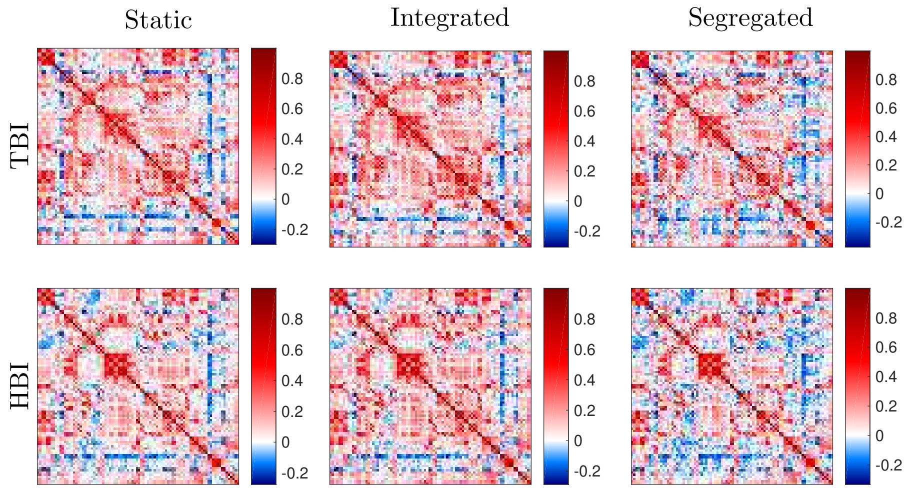

Figure 14: Group-averaged functional connectivity matrices for the static, integrated and segregated states, for DOC patients with traumatic brain injury (TBI, top), and with hypoxic-ischemic brain injury (HBI, bottom). Red indicates a positive value of Pearson correlation, and blue indicates a negative value (anticorrelation). No significant matrix differences between the two aetiologies emerged (two-samples t-tests) after correcting for multiple comparisons, using the Benjamini-Hochberg procedure to control the false discovery rate [3]. Each matrix has been reordered so that ROIs belonging to the same resting-state [2] are adjacent to each other, in the following order: Default mode, visual, somatomotor, salience, dorsal attention, frontoparietal and limbic. Source data are provided as a Source Data file.

Supplementary Tables

Table 1: Brain regions in Montreal Neurological Institute space showing significantly different values of Intrinsic Connectivity Contrast between Awake and Deep anaesthesia.

Contrast	Region Label	Extent	t-value	x	y	z
Positive	Cingulate_Post_L	1059	8.754	-2	-46	28
	Temporal_Pole_Mid_L	154	7.404	-40	16	-36
	Temporal_Mid_L	154	5.398	-56	-6	-22
	SupraMarginal_L	175	6.886	-64	-38	32
	Frontal_Med_Orb_L	61	6.297	-6	52	-12
	Cerebellum_Crus2_L	65	6.279	-34	-70	-38
	Frontal_Sup_Medial_L	159	5.605	0	52	22
	Angular_L	194	5.486	-46	-66	32
	Cerebellum_Crus1_R	78	5.170	34	-86	-34
Negative	Rectus_R	134	-11.804	10	22	-18
	Caudate_R	134	-4.976	10	18	8
	Caudate_L	187	-7.384	-14	20	-2
	Precentral L	284	-6.801	-14	-32	62
	Precentral_R	154	-6.216	28	-16	56
	Postcentral_R	154	-4.191	12	-32	62
	Occipital_Inf_L	93	-5.669	-22	-102	-10
	Postcentral_R	70	-5.335	20	-42	66

Table 2: Brain regions in Montreal Neurological Institute space showing significantly different values of Intrinsic Connectivity Contrast between the conscious healthy controls and DOC patients (conscious > unconscious).

Contrast	Region Label	Extent	t-value	x	y	z
Positive	Cingulate_Post_R	914	7.274	2	-50	26
	Temporal_Mid_L	4440	7.008	-58	-16	-16
	SupraMarginal_L	4440	6.655	-62	-30	28
	Angular_L	4440	6.546	-52	-64	26
	Postcentral_R	1964	6.663	62	-16	32
	Parietal_Inf_R	1964	5.429	58	-54	46
	Temporal_Sup_R	1964	5.074	66	-46	22
	Frontal_Inf_Oper_L	206	6.609	-50	8	12
	Frontal_Sup_Medial_L	264	6.559	-4	68	14
	Frontal_Sup_2_L	264	3.689	-12	60	32
	Temporal Mid R	71	6.054	48	-14	-16
	Frontal_Inf_Oper_R	307	5.500	50	10	12
	Temporal_Pole_Sup_R	307	4.070	52	14	-10
	Frontal_Inf_Tri_L	145	5.127	-40	36	14
	Fusiform_R	76	5.077	38	-72	-14
	Temporal_Inf_R	110	5.008	68	-42	-16
	Temporal_Inf_R	110	3.589	54	-62	-18
	Frontal_Med_Orb_L	160	4.993	-8	54	-6
	Postcentral_L	81	4.941	-58	-8	44
	Frontal_Sup_Medial_L	75	4.930	-4	46	32
	Temporal_Pole_Sup_L	120	4.717	-58	12	-8
	Cerebellum_6_R	71	4.667	24	-72	-18
	Fusiform_R	71	3.534	24	-52	-16
	Parietal_Sup_R	80	4.298	18	-64	64

Table 3: Brain regions in Montreal Neurological Institute space showing significantly different values of Intrinsic Connectivity Contrast between the conscious healthy controls and DOC patients (unconscious > conscious).

Contrast	Region Label	Extent	t-value	x	y	z
Negative	Vermis_4_5	6110	-3.426	-2	-52	-8
	Cerebellum_9_L	6110	-3.445	-12	-46	-40
	Cerebellum_8_R	6110	-3.802	10	-66	-46
	Caudate_R	3802	-3.494	10	20	-4
	Olfactory_R	3802	-9.883	8	26	-12
	Caudate_R	3802	-7.555	22	14	14
	Cingulate_Post_L	6110	-9.388	-12	-44	12
	Vermis_8	6110	-8.122	6	-68	-44
	ParaHippocampal_L	6110	-7.064	-24	-4	-36
	Hippocampus_R	457	-5.700	20	-34	8
	Fusiform_R	457	-4.434	38	-38	-12
	Calcarine_R	457	-3.699	28	-52	12
	Caudate R	72	-5.126	14	-10	22
	Cerebellum_Crus1_L	71	-5.025	-46	-40	-38

Table 4: Brain regions in Montreal Neurological Institute space showing significantly different values in the Sample Entropy of BOLD timeseries between the Awake and Deep anaesthesia conditions.

Contrast	Region Label	Extent	t-value	x	y	z
Positive	Parietal_Inf_L	2495	7.444	-56	-28	48
	Angular_L	2495	5.759	-44	-56	34
	Temporal_Mid_L	2495	4.589	-54	-52	6
	Precuneus_L	2917	7.212	2	-58	30
	Cingulate_Post_R	2917	6.107	4	-36	30
	Cingulate_Mid_L	2917	5.232	-6	-44	50
	Frontal_Inf_Tri_L	1304	5.445	-52	30	14
	Frontal_Mid_2_L	1304	4.443	-48	48	0
	Frontal_Mid_2_L	1304	4.407	-44	14	36

Table 5: Brain regions in Montreal Neurological Institute space showing significantly different values in the Sample Entropy of BOLD timeseries between the conscious healthy controls and DOC patients.

Contrast	Region Label	Extent	t-value	x	y	z
Positive	Cerebellum_4_5_L	25699	9.400	-16	-28	-22
	Precuneus_R	25699	9.366	6	-58	40
	Cerebellum_3_R	25699	8.687	20	-28	-22
	Frontal_Inf_Oper_R	1374	6.761	52	20	30
	Frontal_Mid_2_R	1374	4.661	32	14	62
	Frontal_Inf_Oper_R	1374	4.123	32	20	28
	Temporal_Inf_R	1971	6.147	58	-16	-24
	Temporal_Pole_Sup_R	1971	5.912	56	16	-8
	Postcentral_R	1971	4.473	52	-6	24
	Parietal_Inf_R	2274	5.607	60	-54	42
	Temporal_Mid_R	2274	5.521	60	-52	16
	SupraMarginal_R	2274	4.823	64	-28	38
	Frontal_Sup_2_L	648	5.221	-24	4	70
	Frontal_Mid_2_L	648	4.881	-34	20	60
	Precentral_L	648	4.289	-42	0	62
	Frontal_Sup_Medial_L	820	5.103	-2	60	12
	Frontal_Med_Orb_L	820	4.591	-4	54	-12

Table 6: Statistical comparison of the Sample Entropy of head motion parameters between awake healthy volunteers and DOC patients.

	Awake Mean	Awake SD	DOC Mean	DOC SD	t-value	df	Effect Size	p-value
x	0.43	0.23	0.24	0.15	3.11	37	0.98	0.004**
y	0.36	0.26	0.27	0.17	1.26	37	0.4	0.221
z	0.28	0.18	0.29	0.22	-0.18	37	-0.06	0.857
roll	0.26	0.24	0.22	0.16	0.53	37	0.17	0.593
pitch	0.24	0.18	0.21	0.16	0.56	37	0.18	0.577
yaw	0.15	0.17	0.15	0.11	0.07	37	0.02	0.95

** $p < 0.01$, two-samples t-test. Source data are provided as a Source Data file.

Table 7: Statistical comparison of the Sample Entropy of head motion parameters of healthy volunteers while awake and during deep propofol anaesthesia.

	Awake Mean	Awake SD	Deep Mean	Deep SD	t-value	df	Effect Size	p-value
x	0.43	0.23	0.47	0.27	-0.51	16	-0.17	0.615
y	0.36	0.26	0.49	0.26	-1.66	16	-0.49	0.119
z	0.28	0.18	0.31	0.27	-0.33	16	-0.13	0.746
roll	0.26	0.24	0.41	0.22	-2.29	16	-0.64	0.036
pitch	0.24	0.18	0.33	0.19	-1.67	16	-0.48	0.116
yaw	0.15	0.17	0.41	0.3	-3.23	16	-1.03	0.003**

** $p < 0.01$, repeated-measures t-test. Source data are provided as a Source Data file.

Table 8: Statistical analyses of binarised brain network small-worldness based on the AAL [4] 90-ROI atlas with density ranging between 10 and 25% of connections.

		Awake Mean	Awake SD	Unconscious Mean	Unconscious SD	t-value	df	Effect Size	p-value
Static FC	Awake vs Deep	2.19	0.18	2.07	0.26	1.62	15	0.53	0.125
	Awake vs DOC	2.19	0.18	1.96	0.38	2.23	36	0.72	0.030*
Integrated state	Awake vs Deep	2.08	0.20	1.90	0.26	2.29	15	0.79	0.037*
	Awake vs DOC	2.08	0.20	1.86	0.27	2.87	36	0.92	0.006**
Segregated state	Awake vs Deep	2.07	0.21	2.03	0.23	0.52	15	0.18	0.604
	Awake vs DOC	2.07	0.21	1.91	0.41	1.37	36	0.44	0.180

* $p < 0.05$; ** $p < 0.01$ (repeated-measures t-tests for Awake vs Deep; two-samples t-tests for Awake vs DOC). Source data are provided as a Source Data file.

Table 9: Statistical analyses of weighted brain network small-worldness based on the AAL [4] 90-ROI atlas with density ranging between 30 and 50% of connections.

		Awake Mean	Awake SD	Unconscious Mean	Unconscious SD	t-value	df	Effect Size	p-value
Static FC	Awake vs Deep	1.29	0.08	1.21	0.06	3.01	15	1.13	0.008**
	Awake vs DOC	1.29	0.08	1.17	0.09	4.23	36	1.36	<0.001***
Integrated state	Awake vs Deep	1.26	0.06	1.16	0.06	4.86	15	1.53	<0.001***
	Awake vs DOC	1.26	0.06	1.15	0.09	4.06	36	1.31	<0.001***
Segregated state	Awake vs Deep	1.31	0.08	1.26	0.07	2.22	15	0.69	0.039*
	Awake vs DOC	1.31	0.08	1.21	0.08	3.81	36	1.22	<0.001***

* $p < 0.05$; ** $p < 0.01$; *** $p < 0.001$ (repeated-measures t-tests for Awake vs Deep; two-samples t-tests for Awake vs DOC). Source data are provided as a Source Data file.

Table 10: Statistical analyses of weighted brain network small-worldness based on the Lausanne [5] 234-ROI atlas with density ranging between 10 and 25% of connections.

		Awake Mean	Awake SD	Unconscious Mean	Unconscious SD	t-value	df	Effect Size	p-value
Static FC	Awake vs Deep	1.89	0.20	1.80	0.20	1.48	15	0.42	0.156
	Awake vs DOC	1.89	0.20	1.78	0.26	1.41	36	0.45	0.168
Integrated state	Awake vs Deep	1.87	0.14	1.75	0.19	2.38	15	0.71	0.034*
	Awake vs DOC	1.87	0.14	1.73	0.23	2.16	36	0.70	0.038*
Segregated state	Awake vs Deep	1.76	0.22	1.76	0.16	0.03	15	0.01	0.974
	Awake vs DOC	1.76	0.22	1.74	0.31	0.19	36	0.06	0.848

* $p < 0.05$ (repeated-measures t-tests for Awake vs Deep; two-samples t-tests for Awake vs DOC). Source data are provided as a Source Data file.

Table 11: Entropy of seven well-known resting-state networks derived from static functional connectivity for awake and anaesthetised volunteers.

	Awake Mean	Awake SD	Deep Mean	Deep SD	t-value	df	Effect Size	p-value (corrected)
DMN	0.91	0.02	0.89	0.02	3.25	15	1.24	0.044*
VIS	0.90	0.02	0.89	0.03	1.30	15	0.37	0.336
SOM	0.89	0.03	0.88	0.03	1.22	15	0.40	0.336
SAL	0.90	0.02	0.88	0.02	2.54	15	0.75	0.082
DAN	0.91	0.02	0.91	0.02	-0.14	15	-0.05	0.892
FPN	0.90	0.01	0.91	0.02	-0.23	15	-0.08	0.892
LIM	0.91	0.02	0.90	0.03	1.39	15	0.40	0.336

* $p < 0.05$ (repeated-measures t-tests, FDR-corrected). Source data are provided as a Source Data file.

Table 12: Entropy of seven well-known resting-state networks derived from static functional connectivity for awake volunteers and DOC patients.

	Awake Mean	Awake SD	DOC Mean	DOC SD	t-value	df	Effect Size	p-value (corrected)
DMN	0.91	0.02	0.89	0.02	3.42	36	1.10	0.003**
VIS	0.90	0.02	0.88	0.02	3.25	36	1.04	0.005**
SOM	0.89	0.03	0.87	0.03	2.00	36	0.64	0.065
SAL	0.90	0.02	0.89	0.02	0.42	36	0.13	0.679
DAN	0.91	0.02	0.88	0.02	3.63	36	1.17	0.003**
FPN	0.90	0.01	0.89	0.02	2.51	36	0.81	0.023*
LIM	0.91	0.02	0.88	0.02	5.84	36	1.88	<0.001***

* $p < 0.05$; ** $p < 0.01$; *** $p < 0.001$ (two-samples t-tests, FDR-corrected). Source data are provided as a Source Data file.

Table 13: Entropy of seven well-known resting-state networks derived from the integrated state for awake and anaesthetised volunteers.

	Awake Mean	Awake SD	Deep Mean	Deep SD	t-value	df	Effect Size	p-value (corrected)
DMN	0.91	0.01	0.90	0.01	3.43	15	1.14	0.026*
VIS	0.90	0.02	0.89	0.04	0.44	15	0.15	0.682
SOM	0.90	0.02	0.89	0.02	0.73	15	0.23	0.661
SAL	0.90	0.02	0.89	0.02	2.39	15	0.66	0.107
DAN	0.90	0.01	0.91	0.03	-0.49	15	-0.17	0.682
FPN	0.91	0.01	0.91	0.02	0.98	15	0.28	0.601
LIM	0.92	0.02	0.91	0.02	1.32	15	0.45	0.477

* $p < 0.05$ (repeated-measures t-tests, FDR-corrected). Source data are provided as a Source Data file.

Table 14: Entropy of seven well-known resting-state networks derived from the integrated state for awake volunteers and DOC patients.

	Awake Mean	Awake SD	DOC Mean	DOC SD	t-value	df	Effect Size	p-value (corrected)
DMN	0.91	0.01	0.90	0.01	3.26	36	1.05	0.009**
VIS	0.90	0.02	0.89	0.02	1.52	36	0.49	0.156
SOM	0.90	0.02	0.89	0.02	1.60	36	0.51	0.156
SAL	0.90	0.02	0.90	0.02	1.30	36	0.42	0.205
DAN	0.90	0.01	0.89	0.02	1.61	36	0.52	0.156
FPN	0.91	0.01	0.90	0.02	3.52	36	1.13	0.005**
LIM	0.92	0.02	0.89	0.01	5.59	36	1.80	<0.001***

* $p < 0.05$; ** $p < 0.01$; *** $p < 0.001$ (two-samples t-tests, FDR-corrected). Source data are provided as a Source Data file.

Table 15: Entropy of seven well-known resting-state networks derived from the segregated state for awake and anaesthetised volunteers.

	Awake Mean	Awake SD	Deep Mean	Deep SD	t-value	df	Effect Size	p-value (corrected)
DMN	0.93	0.01	0.92	0.02	2.13	15	0.84	0.336
VIS	0.92	0.01	0.93	0.03	-0.40	15	-0.15	0.732
SOM	0.93	0.02	0.91	0.03	1.56	15	0.55	0.336
SAL	0.93	0.02	0.91	0.02	1.74	15	0.68	0.336
DAN	0.93	0.02	0.93	0.03	0.36	15	0.12	0.732
FPN	0.93	0.01	0.93	0.02	0.60	15	0.23	0.732
LIM	0.93	0.02	0.93	0.02	0.50	15	0.17	0.732

Repeated-measures t-tests, FDR-corrected. Source data are provided as a Source Data file.

Table 16: Entropy of seven well-known resting-state networks derived from the segregated state for awake volunteers and DOC patients.

	Awake Mean	Awake SD	DOC Mean	DOC SD	t-value	df	Effect Size	p-value (corrected)
DMN	0.93	0.01	0.92	0.02	2.79	36	0.90	0.013*
VIS	0.92	0.01	0.91	0.02	2.93	36	0.94	0.013*
SOM	0.93	0.02	0.91	0.02	2.77	36	0.89	0.013*
SAL	0.93	0.02	0.92	0.02	0.32	36	0.10	0.760
DAN	0.93	0.02	0.91	0.02	2.86	36	0.92	0.013*
FPN	0.93	0.01	0.92	0.02	2.04	36	0.66	0.059
LIM	0.93	0.02	0.91	0.02	3.96	36	1.27	0.003**

* $p < 0.05$; ** $p < 0.01$ (two-samples t-tests, FDR-corrected). Source data are provided as a Source Data file.

Table 17: Comparison between DOC patients with traumatic brain injury (TBI) and hypoxic/ischemic brain injury (HBI).

		TBI Mean	TBI SD	HBI Mean	HBI SD	t-value	df	Effect Size	p-value
Static FC	Entropy	0.89	0.01	0.88	0.01	0.34	20	0.14	0.729
	Small-worldness	1.86	0.35	2.07	0.34	-1.44	20	-0.59	0.161
Integrated state	Entropy	0.89	0.01	0.89	0.01	0.35	20	0.14	0.726
	Small-worldness	1.84	0.25	1.92	0.28	-0.74	20	-0.30	0.466
Segregated state	Entropy	0.92	0.01	0.91	0.02	0.12	20	0.05	0.911
	Small-worldness	1.79	0.42	2.01	0.37	-1.30	20	-0.53	0.202
Integration-segregation balance	Integrated state proportion	0.64	0.13	0.62	0.14	0.34	20	0.14	0.746

Two-samples t-tests. Source data are provided as a Source Data file.

References

[1] Lebedev, A. V. *et al.* LSD-induced entropic brain activity predicts subsequent personality change. *Human Brain Mapping* **37**, 3203–3213 (2016). URL `https://onlinelibrary.wiley.com/doi/pdf/10.1002/hbm.23234`. `arXiv:1011.1669v3`.

[2] Yeo, B. T. *et al.* The organization of the human cerebral cortex estimated by intrinsic functional connectivity. *Journal of neurophysiology* **106**, 1125–1165 (2011). URL `http://jn.physiology.org/content/106/3/1125.short`.

[3] Benjamini, Y. & Hochberg, Y. Controlling the false discovery rate: a practical and powerful approach to multiple testing. *Journal of the royal statistical society. Series B (Methodological)* 289–300 (1995).

[4] Tzourio-Mazoyer, N. *et al.* Automated anatomical labeling of activations in spm using a macroscopic anatomical parcellation of the mni mri single-subject brain. *Neuroimage* **15**, 273–289 (2002).

[5] Cammoun, L. *et al.* Mapping the human connectome at multiple scales with diffusion spectrum mri. *Journal of neuroscience methods* **203**, 386–397 (2012).
