# Supplementary material for: Consciousness-specific dynamic interactions of brain integration and functional diversity
Source: Nat Commun. 2019 Oct 10;10:4616. doi: 10.1038/s41467-019-12658-9 (PMC6787094; doi:10.1038/s41467-019-12658-9)
Supplement: Supplementary file 3 — Reporting Summary [file 41467_2019_12658_MOESM3_ESM.pdf]

nature research

Corresponding author(s): Emmanuel A Stamatakis NCOMMS-19-15717A

Last updated by author(s): Sep 11, 2019

Reporting Summary

Nature Research wishes to improve the reproducibility of the work that we publish. This form provides structure for consistency and transparency in reporting. For further information on Nature Research policies, see Authors & Referees and the Editorial Policy Checklist.

Statistics

For all statistical analyses, confirm that the following items are present in the figure legend, table legend, main text, or Methods section.

n/a	Confirmed	
☐	☒	The exact sample size (*n*) for each experimental group/condition, given as a discrete number and unit of measurement
☐	☒	A statement on whether measurements were taken from distinct samples or whether the same sample was measured repeatedly
☐	☒	The statistical test(s) used AND whether they are one- or two-sided<br>*Only common tests should be described solely by name; describe more complex techniques in the Methods section.*
☐	☒	A description of all covariates tested
☐	☒	A description of any assumptions or corrections, such as tests of normality and adjustment for multiple comparisons
☐	☒	A full description of the statistical parameters including central tendency (e.g. means) or other basic estimates (e.g. regression coefficient) AND variation (e.g. standard deviation) or associated estimates of uncertainty (e.g. confidence intervals)
☐	☒	For null hypothesis testing, the test statistic (e.g. *F*, *t*, *r*) with confidence intervals, effect sizes, degrees of freedom and *P* value noted<br>*Give P values as exact values whenever suitable.*
☒	☐	For Bayesian analysis, information on the choice of priors and Markov chain Monte Carlo settings
☒	☐	For hierarchical and complex designs, identification of the appropriate level for tests and full reporting of outcomes
☐	☒	Estimates of effect sizes (e.g. Cohen's *d*, Pearson's *r*), indicating how they were calculated

Our web collection on statistics for biologists contains articles on many of the points above.

Software and code

Policy information about availability of computer code

Data collection	The TIVA Trainer (European Society for Intravenous Aneaesthesia, eurosiva.eu) pharmacokinetic simulation program was used to control the propofol infusion.
Data analysis	Code for the "cartographic profile" (Ref. 25) is freely available online (https://github.com/macshine/integration/).<br>The Brain Connectivity Toolbox code used for graph-theoretical analyses is freely available online (https://sites.google.com/site/bctnet/).<br>The Brain Entropy Toolbox is freely available online (https://cfn. upenn.edu/ zewang/BENtbx.php).<br>The CONN toolbox is freely available online (http://www.nitrc.org/projects/conn).<br>The code used to compute the Sample Entropy (SampEn) of motion timeseries in MATLAB is freely available online (https:// uk.mathworks.com/matlabcentral/fileexchange/35784-sample-entropy).

For manuscripts utilizing custom algorithms or software that are central to the research but not yet described in published literature, software must be made available to editors/reviewers. We strongly encourage code deposition in a community repository (e.g. GitHub). See the Nature Research guidelines for submitting code & software for further information.

Data

Policy information about availability of data

All manuscripts must include a data availability statement. This statement should provide the following information, where applicable:

- Accession codes, unique identifiers, or web links for publicly available datasets
- A list of figures that have associated raw data
- A description of any restrictions on data availability

The data that support the findings of this study are available from the corresponding author upon reasonable request . A Source Data file is also provided for Figures 2 and 3, Supplementary Figures 10-14 and Supplementary Tables 6-17.

Field-specific reporting

Please select the one below that is the best fit for your research. If you are not sure, read the appropriate sections before making your selection.

☐ Life sciences ☒ Behavioural & social sciences ☐ Ecological, evolutionary & environmental sciences

For a reference copy of the document with all sections, see nature.com/documents/nr-reporting-summary-flat.pdf

Behavioural & social sciences study design

All studies must disclose on these points even when the disclosure is negative.

Study description	This study uses quantitative methods.
Research sample	Anaesthesia dataset: 19 healthy volunteers (native English speakers, right-handed; 18–40 years; 13 males) . DOC patients: 71 patients meeting diagnostic criteria for Unresponsive Wakefulness Syndrome/Vegetative State or Minimally Conscious State were initially recruited for the study, and the final cohort analysed in this study comprised 22 individuals (14 males; 17 -70 years; mean time post injury: 13 months; 10 UWS, 12 MCS).
Sampling strategy	No power analysis was performed prior to data collection; both final sample sizes are within the range reported in the literature.
Data collection	For the DOC dataset, in addition to the researcher and radiographer, a research nurse was also present. Since the patients' status as DOC patients was evident, no researcher blinding was possible.<br>For the propofol dataset, two senior anaesthesiologists and one anaesthesia nurse were also present, and they could not be blinded to experimental condition, since part of their role involved determining the participants' level of anaesthesia. Propofol was administered intravenously using an Baxter AS50 auto syringe infusion pump (Baxter Healthcare, Singapore).
Timing	DOC patient data were collected between January 2010 and December 2015; propofol data were collected between May and November 2014.
Data exclusions	Due to equipment malfunction or physiological impediments to anaesthesia in the scanner, data from three healthy volunteers (1 male) from the propofol dataset were excluded from analyses, leaving 16 volunteers for analysis.<br>Out of 71 DOC patients recruited for the study, individuals were systematically excluded from the final cohort analysed in this study based on the following criteria: 1) large focal brain damage (i.e. more than 1/3 of one hemisphere) as stated by an expert in neuroanatomy blinded to the patients' diagnoses; 2) excessive head motion during resting state scaning (i.e. greater than 3mm in translation and/or 3 degrees in rotation); 3) suboptimal segmentation and normalization of images. Thus, the final cohort analysed in this study comprised 22 patients (14 males; 17 -70 years; mean time post injury: 13 months; 10 UWS, 12 MCS).
Non-participation	No participants declined participation.
Randomization	No randomisation was present: for the anaesthesia dataset, all participants were run in both conditions (awake and anaesthetised) since this was a repeated measures design. The DOC patients were drawn from the local patient population; to exclude spurious results from the comparison between awake volunteers and DOC patients that were due to differences of no interest between the datasets, only results that were common to both comparisons (awake vs DOC and awake vs anaesthetised) were considered.

Reporting for specific materials, systems and methods

We require information from authors about some types of materials, experimental systems and methods used in many studies. Here, indicate whether each material, system or method listed is relevant to your study. If you are not sure if a list item applies to your research, read the appropriate section before selecting a response.

Materials & experimental systems

n/a	Involved in the study	
☒	☐	Antibodies
☒	☐	Eukaryotic cell lines
☒	☐	Palaeontology
☒	☐	Animals and other organisms
☐	☒	Human research participants
☒	☐	Clinical data

Methods

n/a	Involved in the study	
☒	☐	ChIP-seq
☒	☐	Flow cytometry
☐	☒	MRI-based neuroimaging

Human research participants

Policy information about [studies involving human research participants](#)

Population characteristics	See above.
Recruitment	For the anaesthesia study, we recruited participants with posters around campus as per ethics protocol. Participants approaches the research team to seek participation, and there were no specific selection biases. Participants were required to be healthy, right-handed, native English speakers with no history of neurological disorders, and no contraindications to MRI scanning.<br>DOC patients were recruited from specialised long-term care centres. To be invited to the study, patients must have had a DOC diagnosis, written informed consent to participation from their legal representative, and were capable of being transported to Addenbrooke's Hospital. The exclusion criteria included any medical condition that made it unsafe for the patient to participate (decision made by clinical personnel blinded to the specific aims of the study) or any reason they are unsuitable to enter the MRI scanner environment (e.g. non-MRI-safe implants), significant pre-existing mental health problems, or insufficient English pre-injury.
Ethics oversight	Ethical approval for testing DOC patients was provided by the National Research Ethics Service (National Health Service, UK; LREC reference 99/391). For the anaesthesia dataset, the Health Sciences Research Ethics Board and Psychology Research Ethics Board of Western University (Ontario, Canada) ethically approved the study.

Note that full information on the approval of the study protocol must also be provided in the manuscript.

Magnetic resonance imaging

Experimental design

Design type	Resting-state.
Design specifications	DOC dataset: 10 minutes continuous scan; anaesthesia dataset: 8 minutes continuous scan.
Behavioral performance measures	In both datasets, lack of behavioural responsiveness was used as an indicator of unconsciousness.<br>For the DOC patients, it was assessed clinically at multiple times over the period of their stay in Addenbrooke's Hospital (Cambridge, UK).<br>For the propofol dataset, failure to perform two computerised tasks (a computerised auditory target-detection task and a memory test of verbal recall) was used to evaluate the level of wakefulness in the anaesthesia condition independently of the assessors, who also evaluated participants' level of behavioural responsiveness based on the Ramsay scale.

Acquisition

Imaging type(s)	Functional and anatomical.
Field strength	3T for both datasets.
Sequence & imaging parameters	Anaesthesia dataset: MRI scanning was performed using a 3-Tesla Siemens Tim Trio scanner (32-channel coil), and 256 functional volumes (echo-planar images, EPI) were collected from each participant, with the following parameters: slices = 33, with 25% inter-slice gap; resolution = 3mm isotropic; TR = 2000ms; TE = 30ms; flip angle = 75 degrees; matrix size = 64x64. The order of acquisition was interleaved, bottom-up. Anatomical scanning was also performed, acquiring a high-resolution T1- weighted volume (32-channel coil, 1mm isotropic voxel size) with a 3D MPRAGE sequence, using the following parameters: TA = 5min, TE = 4.25ms, 240x256 matrix size, 9 degrees FA.<br><br>DOC patients: Resting-state fMRI was acquired for 10 minutes (300 volumes, TR=2000ms) using a Siemens Trio 3T scanner (Erlangen, Germany). Functional images (32 slices) were acquired using an echo planar sequence, with the following parameters: 3 x 3 x 3.75mm resolution, TR = 2000ms, TE = 30ms, 78 degrees FA. Anatomical scanning was also performed, acquiring high-resolution T1-weighted images with an MPRAGE sequence, using the following parameters: TR = 2300ms, TE = 2.47ms, 150 slices, resolution 1 x 1 x 1mm.
Area of acquisition	Whole brain scan.

Diffusion MRI ☐ Used ☒ Not used

Preprocessing

Preprocessing software	We used the CONN toolbox version 17f, based on SPM12. The default pipeline was used. The pipeline comprised the following steps: removal of the first five scans, to allow magnetisation to reach steady state; functional realignment and motion correction; slice-timing correction to account for differences in time of acquisition between slices; identification of outlier scans by means of the quality assurance/artifact rejection software ART; spatial normalisation to Montreal Neurological Institute (MNI-152) standard space with 2mm isotropic resampling resolution, using the segmented grey matter image from each volunteer's high-resolution T1-weighted image, together with an a priori grey matter template; spatial smoothing with a Gaussian kernel of 6mm full width at half-maximum (FWHM).<br>Due to the presence of deformations caused by brain injury, rather than relying on automated pipelines, DOC patients'

brains were individually preprocessed using SPM12, with visual inspections after each step, and subsequently denoised following the steps outlined above within the CONN toolbox.

Normalization

Direct normalisation to MNI space (nonlinear) using the segmented grey matter image from each volunteer's high-resolution T1-weighted image, together with an a priori grey matter template.

Normalization template

MNI152 template.

Noise and artifact removal

The anatomical CompCor method was used, regressing out of the functional data the following confounding effects: the first five principal components attributable to each individual's white matter signal, and the first five components attributable to individual cerebrospinal fluid (CSF) signal; six subject-specific realignment parameters (three translations and three rotations) as well as their first- order temporal derivatives; the nuisance regressors identified by ART; and main effect of scanning condition. Linear detrending was also applied, and the subject-specific denoised BOLD signal timeseries were band-pass filtered to eliminate both low-frequency drift effects and high-frequency noise, thus retaining frequencies between 0.008 and 0.09 Hz. For the DOC patients, data underwent additional despiking with a hyperbolic tangent squashing function.

Volume censoring

The artifact rejection tool (ART), implemented in the CONN toolbox, was used to identify and regress out outlying volumes, as part of the CompCor denoising procedure described above. The default CONN settings of 5 global signal z-values and 0.9mm were used. For the awake healthy volunteers, this resulted in an average of 1% of volumes being identified as outliers; in the anaesthetised condition, a mean of 10% of volumes were identified as artifactual. For the DOC patients, the mean percentage of volumes identified as artifacts was 11%.

Statistical modeling & inference

Model type and settings

Mass univariate model; two-samples design for the comparison between DOC patients and healthy volunteers, and repeated-measures for comparing the healthy volunteers when awake and when anaesthetised. All tests were two-sided. Effect sizes were measured using Hedge's g statistic.

Effect(s) tested

Voxelwise , the Intrinsic Connectivity Contrast and Sample Entropy were compared. Follow-up analyses were performed using seed-based connectivity. Group-level comparisons were implemented as a GLM.
Connectivity entropy and network small-worldness of different temporal states were also compared across states of consciousness; connectivity entropy was assessed both for the whole brain and for individual resting-state networks.

Specify type of analysis: ☐ Whole brain ☐ ROI-based ☒ Both

Anatomical location(s)

The seeds for the seed-based correlation were determined from the outputs of the Intrinsic Connectivity Contrast analysis. ROIs for the construction of the connectivity matrices were derived from the AAL atlas, excluding cerebellum and vermis (total of 90 ROIs). The Lausanne atlas with 234 ROIs was also used to ensure robustness of our findings.

Statistic type for inference
(See Eklund et al. 2016)

Output maps of group differences were thresholded at voxelwise $p < 0.001$ (uncorrected), and corrected for multiple comparisons by applying a family-wise error (FWE) cluster-based correction, resulting in $p < 0.05$.

Correction

FWE correction.

Models & analysis

n/a	Involved in the study
☐	☒ Functional and/or effective connectivity
☐	☒ Graph analysis
☒	☐ Multivariate modeling or predictive analysis

Functional and/or effective connectivity

Dynamic connectivity matrices were derived using an overlapping sliding- window approach[11,25]. For each subject and each condition, tapered sliding windows were obtained by convolving a rectangle of 22 TRs (44s) with a Gaussian kernel of 3 TRs, sliding with 1 TR step size. Within each of the resulting overlapping temporal windows of 22 TRs, a 90-by-90 matrix of functional connectivity was estimated, with the connection between each pair of AAL-derived ROIs being given by the Pearson correlation between their timecourses within that window.

Graph analysis

Following Shine et al., (2016) and Fukushima et al., (2018), states of higher integration or segregation were identified from the connectivity between regions, by establishing a "cartographic profile" based on the module assignments of each ROI, considered as a network node. Modules in each time- resolved functional connectivity matrix were identified by means of the Louvain greedy algorithm for maximisation of the modularity function Q[68], accounting for both positive and negative connections using the asymmetric algorithm of Rubinov and Sporns (2011)[67] (Eq. 3). In the case of signed graphs, a module is defined as a group of nodes that are positively correlated with each other, but negatively correlated with nodes belonging to different modules [8]. Due to its stochastic nature, the algorithm was repeated for 100 iterations for each time- resolved network, and the module size resolution parameter gamma was set to one, the default [25,26]. Based on the modularity assignments, we then derived the participation coefficient and within-degree Z-score for each node (see Eq. 4 and 5). Subsequently, joint histograms of participation coefficient and within-module Z-score were produced for each timepoint[25], since together,

these two measures quantify both a node's inter modular and intramodular connectivity. For each subject, the joint patterns were then used to assign each timepoint to one of two clusters, using an unsupervised machine learning algorithm known as k-means clustering (setting k = 2)[25]. To avoid the possibility of the algorithm becoming stuck in local minima, it was repeated 500 times with random re-initialisation of the two clusters' initial points. This was performed individually for each subject and condition. Following Shine et al (2016), Pearson correlation was chosen as distance metric for the algorithm.
Finally, the cluster with higher mean participation coefficient was labelled as the integrated state, while the cluster with lower average participation coefficient was considered to be the segregated state[25]. For each subject, a centroid matrix of functional connectivity was computed for each state, as the element-wise median of the timepoint-specific FC matrices assigned to the cluster corresponding to that state. The proportion of time spent in each state was also estimated, as the number of timepoints assigned to that cluster, over the total number of timepoints.
For each state (integrated, segregated, and also static functional connectivity for comparison), the global measure of small-worldness was used as DV, and compared at the group level. State-specific matrices of functional connectivity were thresholded proportionally for each participant and condition to retain the strongest positive connections, using a range of thresholds. Results were obtained separately at each threshold level and then averaged together before statistical comparison. To ensure robustness of the results, analyses were performed on both weighted and binarised graphs, and using different threshold ranges.
